# Fingolimod (FTY720) Stimulates Ca^2+^/Calcineurin Signaling in Fission Yeast

**DOI:** 10.1371/journal.pone.0081907

**Published:** 2013-12-03

**Authors:** Kanako Hagihara, Ayako Kita, Aya Mizukura, Mariko Yao, Yuki Kitai, Tatsuki Kunoh, Takashi Masuko, Sumio Matzno, Kenji Chiba, Reiko Sugiura

**Affiliations:** 1 Laboratory of Molecular Pharmacogenomics, School of Pharmaceutical Sciences, Kinki University, Higashi-osaka, Japan; 2 Laboratory of Molecular Cell Biology, School of Pharmaceutical Sciences, Kinki University, Higashi-osaka, Japan; 3 Division of Pharmaceutical Education, Kinki University Faculty of Pharmacy 3-4-1, Kowakae, Higashi-Osaka, Osaka, Japan; 4 Advanced Medical Research Laboratories, Research Division, Mitsubishi Tanabe Pharma Corporation, Yokohama, Japan; 5 Research Fellow of Japan Society for the Promotion of Science, 1-8 Chiyoda-ku, Tokyo, Japan; Temple University School of Medicine, United States of America

## Abstract

Fingolimod hydrochloride (FTY720) is the first in class of sphingosine 1-phosphate (S1P) receptor modulator approved to treat multiple sclerosis via down-regulation of G protein-coupled S1P receptor 1 by its phosphorylated form (FTY720-P). Many studies have revealed that FTY720 exerts various biological effects, including antitumor activities, angiogenesis inhibition, Ca^2+^ mobilization and apoptosis, independently of S1P receptors. However, the exact mechanisms underlying their effects or signaling pathways mediated by FTY720 have not been completely established. To gain further insights into molecular mechanisms of FTY720 action, the effect of FTY720 on Ca^2+^ signaling in fission yeast was analyzed. The addition of Ca^2+^ enhanced the sensitivity induced by FTY720, and mutants lacking genes required for calcium homeostasis, including calcineurin and its downstream transcription factor, Ppb1-responsive zinc finger protein (Prz1), were hypersensitive to FTY720 and CaCl_2_. The effect of FTY720 on calcineurin signaling was monitored by utilizing a luciferase reporter construct fused to three tandem repeats of the calcineurin-dependent response element (CDRE), which gives an accurate measure of calcineurin activity. The addition of FTY720 increased calcineurin activity as well as Ca^2+^ influx in a concentration-dependent manner. Notably, the FTY720-mediated Ca^2+^ influx and calcineurin activation were reduced markedly by the deletion of *yam8* ^+^ or *cch1* ^+^ encoding putative subunits of a Ca^2+^ channel. Consistently, the deletion of Pmk1 mitogen-activated protein kinase (MAPK), which plays an important role in the activation of the Yam8/Cch1 channel, markedly decreased the intracellular Ca^2+^ levels upon FTY720 treatment. These results suggest that the FTY720-stimulated Ca^2+^/calcineurin signaling activation partly involves the Yam8/Cch1 channel in fission yeast.

## Introduction

Fingolimod hydrochloride (FTY720) is a novel sphingosine 1-phophate (S1P) receptor modulator that was found by chemical modification of myriocin, a natural product isolated from culture filtrates of the ascomycete *Isaria sinclairii* [1]. FTY720 inhibits lymphocyte egress from lymph nodes to efferent lymphatics and blood, and the immunomodulating effects of FTY720 are largely elicited following its phosphorylation by sphingosine kinase (SphK)2 and the subsequent modulation of G protein-coupled S1P receptor 1 [2,3]. Although the biological effects of FTY720 have been generally attributed to its actions as an S1P mimetic upon its phosphorylation, considerable evidence suggests that FTY720 may act through more than one target.

Interestingly, in addition to its therapeutic use as an immunomodulating drug, FTY720 was also shown to exert potent antitumor and antimetastatic activities in different tumor types, including breast cancer, bladder cancer, hepatocellular carcinoma, and leukemia [4,5]. Several hypotheses explain the antitumor activity of FTY720. Reports have shown that FTY720 induced the mitochondrial permeability transition and consequent activation of caspases, with the modulation of these processes by the mitochondrial gatekeeper Bcl-2 family proteins [6,7]. FTY720 is also known to downregulate prosurvival mitogen-activated protein kinase (MAPK) and phosphatidylinositol 3-kinase/Akt pathways and upregulate stress-activated kinases such as p38 [8,9]. FTY720 also increases the intracellular concentration of calcium ions and induces apoptosis in HL-60 [10]. 

Accumulating evidence also suggests that FTY720 may exert some of these effects independently of S1P receptors by modulating a range of other recently described proteins targeted by nonphosphorylated FTY720 [11]. For example, FTY720 inhibits cytosolic phospholipase A2 independently of its phosphorylation and S1P receptor functions [12]. However, although diverse physiological and therapeutic effects have been documented for this compound, the multifaceted mechanism of the action of FTY720 remains unclear. 

This study uses fission yeast as a model eukaryotic system to dissect the biological activity of FTY720. The fission yeast *Schizosaccharomyces pombe* and the budding yeast *Saccharomyces cerevisiae* are among the simplest eukaryotic organisms that are widely used as valuable tools for the study of basic cellular processes and pathways relevant to higher eukaryotes, including mechanisms of cell cycle control, metabolism, and membrane trafficking [13,14]. Both these strains are also excellent organisms for the identification of molecular targets and elucidation of molecular/cellular mechanisms of sensitivity to various drugs because the major signaling pathways and processes involved in the cellular response to cytotoxic agents are conserved between yeasts and mammalian cells [15-18]. In budding yeast, it has been reported that ubiquitin pathway proteins are involved in the mechanism of action of FTY720 [19] and that FTY20 and phytosphingosine influence a similar pathway in yeast cells [20].

To better understand the signaling pathways mediated by FTY720, the effect of FTY720 on Ca^2+^/calcineurin signaling was analyzed. In fission yeast, a mutation in *ppb1* ^+^ encoding the Ca^2+^/calmodulin-dependent protein phosphatase [17,21] as well as a mutation in *prz1* ^+^ encoding a transcription factor downstream of calcineurin involved in Ca^2+^ homeostasis [22] affected the sensitivity of cells to FTY720, suggesting that a disturbance in Ca^2+^ homeostasis results in increased sensitivity to FTY720. We also demonstrated that FTY720 activates calcineurin signaling by stimulating Ca^2+^ influx mediated by the Yam8/Cch1 Ca^2+^ channel in fission yeast. 

## Materials and Methods

### Strains, Media, and Genetic/Molecular Biology Methods

The S. *pombe* strains used in this study are listed in Table 1. The complete and minimal media used were yeast extract-peptone-dextrose (YPD) and Edinburgh minimal medium (EMM) [23], respectively. Standard genetic and recombinant DNA methods [24] were used, except where stated otherwise. Gene knockouts are denoted by lowercase letters representing the disrupted gene, followed by two colons and the wild-type (wt) gene marker used for the disruption (e.g., *ppb1::ura4* ^*+*^). In addition, gene knockouts are abbreviated by the gene, which is preceded by Δ (e.g., Δ*ppb1*). Proteins are denoted by Roman letters, and only the first letter is capitalized (e.g., Ppb1). Genome DNA clones were provided by the National Bio Resource Project, Yeast Genetic Resource Center (Graduate School of Science, Osaka City University). 

**Table 1:** Schizosaccharomyces pombe strains used in this study.

Strain	Genotype	Reference
HM123	*h^-^ leu1-32*	Our stock
KP119	*h^+^ leu1-32 ura4-D18 ppb1::ura4**^+^***	Ma et al., (2011)
KP1003	*h ^-^leu1-32 ura4-D18 prz1::ura4**^+^***	Hirayama et al., (2003)
KP2758	*h^-^ leu1-32 ura4-D18 yam8::ura4**^+^***	Deng et al., (2006)
KP2784	*h^-^ leu1-32 ura4-D18 cch1::ura4**^+^***	Deng et al., (2006)
SP983	*h* ^-^ *leu1-32 ura4-D18 yam8::*KanMX6 *cch1::ura4* **^*+*^**	Our stock
SP385	*h* ^–^ *leu1-32 ura4-D18 pmk1*::KanMX6	Bimbó et al., (2005) [44]
SP1937	*h* ^–^ *leu1-32 ura4-D18 bfr1::ura4* **^*+*^** * pmd1*::*hisG*	kind gift from Minoru Yoshida

### Chemicals

FTY720 and FTY720-P (phosphorylated FTY720) were kindly received as gifts from the Mitsubishi Tanabe Pharma Corporation (Yokohama, Japan). Yeast growth media containing each of these chemicals were prepared by mixing stock solutions of FTY720 or FTY720-P with the YPD or YES medium to achieve the desired drug concentration. For agar media, the stock solution of the appropriate drug was added after autoclaving and cooling of the media to approximately 50°C. For FTY720, the stock solution was prepared using water, whereas for FTY720-P, the stock solution was prepared using 80% ethanol containing 10 mM NaOH [25]. When comparing the effects of FTY720 and FTY720-P, FTY720 was dissolved in the same vehicle as FTY720-P to unify the basal conditions. Each vehicle was added to control media at concentrations equivalent to that in the media supplemented with FTY720 or FTY720-P.

### Microscopy and Miscellaneous Methods

Light microscopy methods (e.g., fluorescence microscopy) used to observe the localization of GFP-Prz1 was performed as described previously [26]. 

### Image Quantification

All the image quantifications were performed for three individual datasets, which summed up to 300 counted cells. 

### Statistical Analysis

The mean and the standard deviation (SD) of the relative light units (RLU) values for three different experiments were calculated for each sample. Data were analyzed using a two-way ANOVA, followed by a posthoc test using Williams’ multiple comparison.

### Live Cell Monitoring of Calcineurin-mediated Transcriptional Activity

Calcineurin-dependent response element (CDRE)-reporter activity in living cells was monitored by the multicopy 3×CDRE::luc (R2.2) reporter vector pKB5723 as described previously [27]. We also created the mutant version of the 3×CDRE::luc (R2.2) reporter vector by replacing the wt CDRE oligonucleotide (AGCCTC or GAGGCT) in pKB5723 with the mutant CDRE oligonucleotide (sense 884: 5′-GGC TTA GAT CTA TAC AAG ATC TAT ACA CAA GAT CTA TGCAC-3′; antisense 885: 5′-TCG AGT GCA TAG ATC TTG TGT ATA GAT CTT GTA TAG ATC TAA GCC TGCA-3′) that contains three tandem repeats of the mutated CDRE (AGATCT, underlined), thereby yielding the multicopy 3×mtCDRE::luc(R2.2) reporter vector pKD2767. 

### Measurement of Cytoplasmic Ca^2+^ Levels

Cytoplasmic Ca^2+^ levels were determined using a previously described method with minor modifications [28]. In brief, cells containing GFP-19-apoaequorin (AEQ) were grown at 27°C in the EMM medium and harvested (to midlog phase) in the early logarithmic growth phase. The cells were resuspended in fresh EMM containing 10 μM coelenterazine (Promega), and the optical density was adjusted to 0.6 at 660 nm. To convert AEQ to aequorin, the cells were incubated for 3 h at 27°C. The cells were washed twice, resuspended in fresh EMM, and the optical density was adjusted to 0.6 at 660 nm. The cell culture was then incubated at 27°C for 30 min before initiating the experiment. The light emission levels expressed as RLU were measured using a luminometer (Centro XS^3^ LB960: Berthold) at 30-s intervals.

## Results

### FTY720 inhibits growth of *S. pombe.*

**Figure 1:**
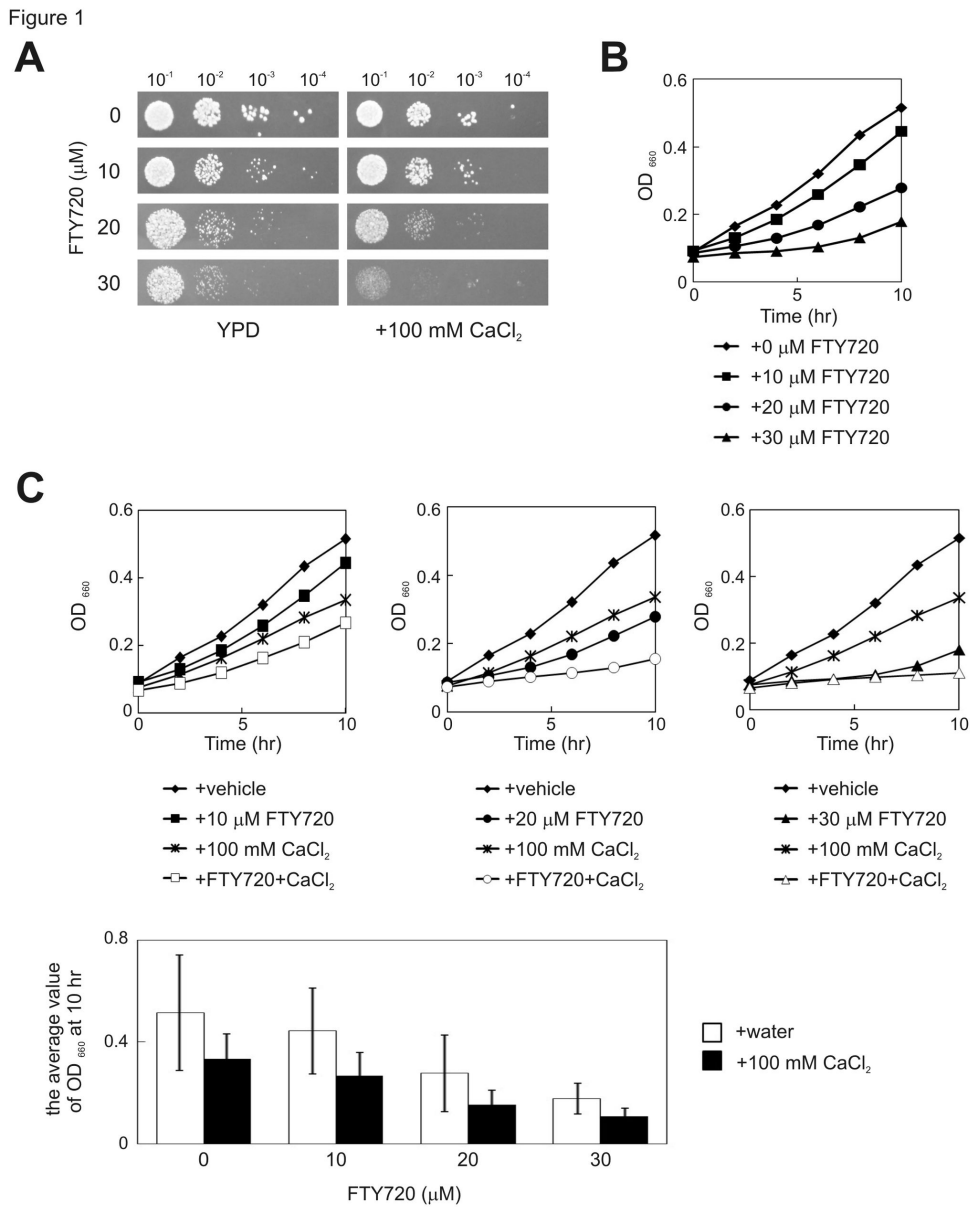
Fission yeast sensitivity to FTY720. (A) Fission yeast cells are sensitive to FTY720. A serial dilution assay of the wild-type strain grown in YPD medium or YPD medium containing the indicated concentrations of FTY720 in the absence (Left: YPD) or presence (Right: + 100 mM CaCl_2_) of 100 mM CaCl_2_. 　Cells were incubated for 3 days at 27°C. (B) Quantitative measurements of cell growth in the presence of FTY720. The cells were grown in liquid YES cultures to an OD_660_ of 0.3 and were treated with the drugs (FTY720) at the concentrations indicated, and the quantitative measurements of cell growth rates were performed using a microplate reader (Sunrise^TM^ series, Tecan, Switzerland). A representative for three independent curves is presented. (C) Addition of CaCl_2_ exacerbated the fission yeast sensitivity to FTY720. Wild-type cells were cultured in YES liquid medium and treated with 100 mM CaCl_2_ in the absence or presence of indicated concentrations of FTY720, and the growth curve of the cells were shown by measuring OD_660_ for 10 h. (D) Graph shows the OD_660_ at 10 h of the cells, as indicated in Figure 1(B) and (C). The data were averaged from three independent experiments. Bars, SD.

FTY720 was tested at a range of concentrations and found to inhibit growth of wt cells in a dose-dependent manner (Figure 1A). To quantitatively measure the effect of FTY720, the influence of the compound on the growth of wt cells in the liquid medium was assessed, and dose-dependent growth inhibition induced by FTY720 was confirmed (Figure 1B). To analyze the relationship between Ca^2+^ signaling and FTY720 sensitivity, the effect of Ca^2+^ on the growth of wt cells in the medium supplemented with FTY720 was first tested. As shown in Figure 1A, the addition of 100 mM CaCl_2_ alone only marginally affected the growth of wt cells in the solid YPD medium. However, the same concentration of CaCl_2_ exacerbated the growth of wt cells treated with various concentrations of FTY720 (Figure 1A). The effect of Ca^2+^ on FTY720-induced cell growth defects was also confirmed by measuring the OD at 660 nm in a microplate reader (Figure 1C).

### Fission yeast mutants with defects in Ca^2+^/calcineurin signaling are hypersensitive to FTY720.

**Figure 2:**
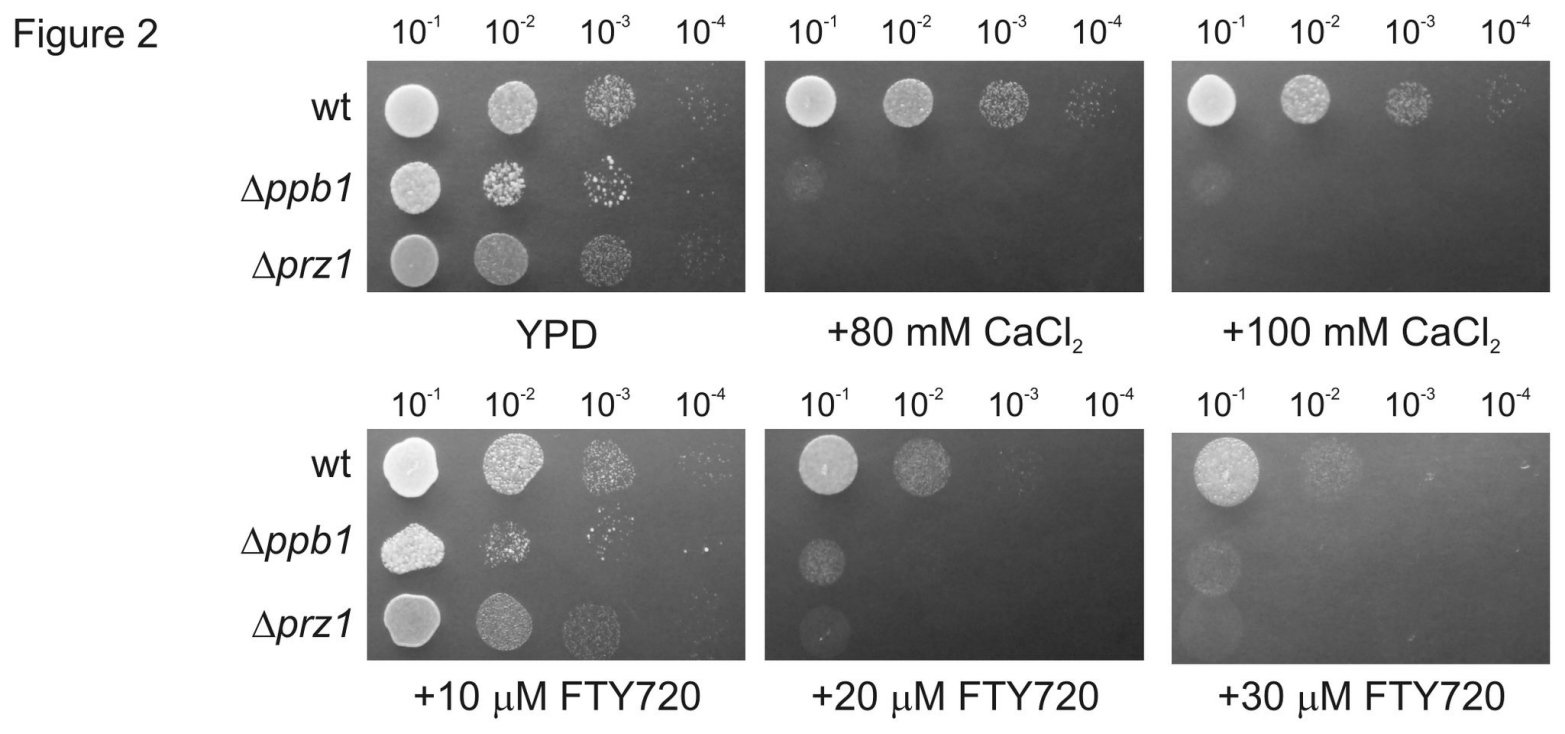
Defects in calcineurin signaling caused hypersensitivity to FTY720. A serial dilution assay of the wild-type (wt), Δ*ppb1*, and Δ*prz1* cells grown in rich YPD medium containing the indicated concentrations of FTY720 or CaCl_2_.

The effect of mutated Ca^2+^ signaling on FTY720 sensitivity was next examined. The addition of 10 μM FTY720 only slightly inhibited the growth of wt cells (Figure 2, +10μM FTY720, wt). In contrast, knockout of *ppb1* ^+^, which encodes the Ca^2+^/calmodulin activated protein phosphatase [28], resulted in significant sensitivity to the same concentration of FTY720. The Δ*ppb1* cells almost failed to grow in the presence of 30 μM FTY720, whereas the wt cells formed colonies (Figure 2, Δ*ppb1*). In fission yeast, calcineurin dephosphorylates and activates Prz1, a zinc finger-type transcription factor involved in Ca^2+^ homeostasis [26]. Deletion of Prz1 also enhanced the sensitivity to FTY720 because Δ*prz1* cells failed to grow in media containing more than 20 μM FTY720 (Figure 2, Δ*prz1*). Notably, these mutant cells also displayed enhanced sensitivities to CaCl_2_ compared with the wt cells because they failed to grow in the medium containing more than 80 mM CaCl_2_ (Figure 2). These results suggest that the mechanism underlying FTY720 sensitivity may involve impaired Ca^2+^ signaling. 

### FTY720 stimulates the calcineurin/Prz1 signaling pathway.

**Figure 3:**
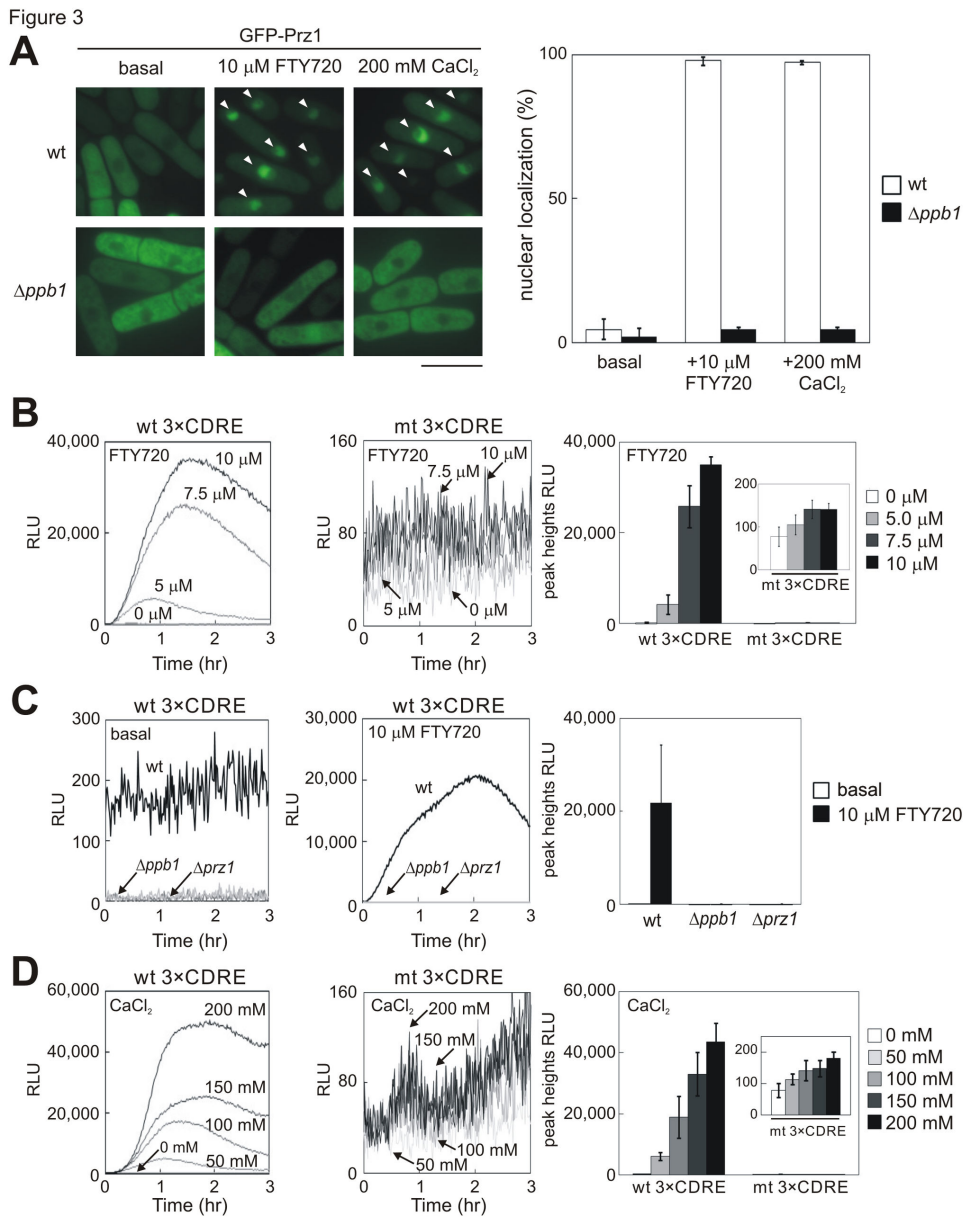
FTY720 stimulates the calcineurin/Prz1 signaling pathway. (A) Left: Translocation of GFP-Prz1 to the nucleus is induced by FTY720 addition and requires calcineurin. Wild-type (wt), or calcineurin-null cells (Δ*ppb1*) expressing GFP-Prz1 were grown in EMM medium at 27°C and analyzed by fluorescence microscopy to observe GFP-Prz1 localization (GFP-Prz1). The cells were incubated with or without 10 μM FTY720 for 10 min or in the presence of 200 mM CaCl_2_ for 10 min at 27 °C.  Arrowheads indicate cells whose nuclei show intense GFP fluorescence. The bar indicates 10 μm. Right: The percentage of cells in (A) showing intense nuclear fluorescence of GFP-Prz1 was measured. At least 300 cells were counted. (B) Real-time monitoring of calcineurin activity in living cells stimulated by FTY720. Wild-type cells harboring the multicopy plasmid wt 3×CDRE::luc(R2.2) reporter vector pKB5723 (wt 3×CDRE) or the multicopy plasmid carrying the mutant version of the CDRE (mt 3×CDRE::luc(R2.2)) reporter vector pKD2767 (mt 3×CDRE) were incubated with D-luciferin and treated with various concentrations of FTY720, as indicated. Using a luminometer, light emission levels expressed as relative light units (RLU) were measured per minutes for 3 h. The data shown are representative of multiple experiments. Right: Graph shows the peak heights of 3×CDRE::luc(R2.2) (wt 3×CDRE) or the mutant 3×CDRE::luc(R2.2) (mt 3×CDRE) reporter activity. The data were averaged from three independent experiments. Bars, SD. (C) Stimulation of calcineurin signaling induced by FTY720 addition requires calcineurin/Prz1 signaling. Wild-type and *ppb1*─ and *prz1*─null cells harboring the multicopy plasmid (wt 3×CDRE::luc(R2.2)) reporter vector were treated with 10 μM FTY720 and monitored as described in Figure 3B. (D) Real-time monitoring of calcineurin activity in living cells stimulated by CaCl_2_. Cells as indicated in Figure 3 (B) were treated with various concentrations of CaCl_2_, as indicated, and monitored as described in Figure 3 (B).

The above findings prompted the examination of the effect of FTY720 on calcineurin/Prz1 signaling. The intracellular localization of GFP-Prz1 was examined because the activation of calcineurin causes the translocation of GFP-Prz1 from the cytoplasm to the nucleus [26]. The addition of 10 μM FTY720 stimulated the nuclear accumulation of GFP-Prz1 because more than 95% of wt cells exhibited nuclear localization of GFP-Prz1 (Figure 3A, wt, +10 μM FTY720, arrowheads). On the other hand, in calcineurin-null cells treated with the same concentration of FTY720, GFP-Prz1 almost remained cytosolic, with less than 5% of the cells localizing to the nucleus (Figure 3A, Δ*ppb1*, +10 μM FTY720). The addition of 200 mM CaCl_2_ exerted similar effects on GFP-Prz1 localization (Figure 3A, +200 mM CaCl_2_, arrowheads). Thus, FTY720 stimulates nuclear translocation of Prz1 as efficiently as Ca^2+^.

The effect of FTY720 on calcineurin/Prz1 signaling was then further tested using the reporter construct 3×CDRE fused to R2.2 destabilized luciferase (3×CDRE::luc(R2.2)), which was established as an accurate measure of the calcineurin activation in living cells [27]. The wt cells harboring the reporter plasmid 3×CDRE::luc(R2.2) were stimulated with various concentrations of FTY720 (5.0–10 μM) or CaCl_2_ (50–200 mM). FTY720 treatment caused a dose-dependent increase in the 3×CDRE::luc (R2.2) response, exhibiting a peak rise and then approaching a constant level (Figure 3B, wt 3×CDRE). On the other hand, in calcineurin-null (Δ*ppb1*) and *prz1*-null (Δ*prz1*) cells treated with 10 μM FTY720, the reporter response both in the absence (basal) and the presence of FTY720 was reduced markedly (Figure 3C), consistent with the notion that the 3×CDRE reporter gives an accurate measure of the calcineurin/Prz1 system. The reporter response obtained by FTY720 treatment was similar to that obtained by CaCl_2_ addition (Figure 3D, wt 3×CDRE). Thus, FTY720 as well as Ca^2+^ stimulates calcineurin/Prz1 signaling.

The mutant version of the 3×CDRE reporter plasmid 3×mt CDRE::luc (R2.2) was next created, in which the consensus motif AGCCTC in the CDRE was mutated to AGATCT (Materials and Methods). The mutation in CDRE dramatically decreased the reporter activity upon FTY720 and CaCl_2_ treatment (Figure 3B, D, mt 3×CDRE), confirming that the above mentioned reporter activity stimulated by FTY720 and CaCl_2_ was exerted via Prz1 binding to the CDRE motif.

While this range of concentration is higher than that required for immune modulation (10-100 nM) in cultured mammalian cells, it should be noted that significantly higher concentrations of FTY720 were used (5-25 μM) when examining the inhibition of tumor development, induction of apoptosis and stimulation of the intracellular concentration of calcium ions in mammalian cells [4,9,10,29-32]. 

### FTY720 stimulates Ca^2+^ influx.

**Figure 4:**
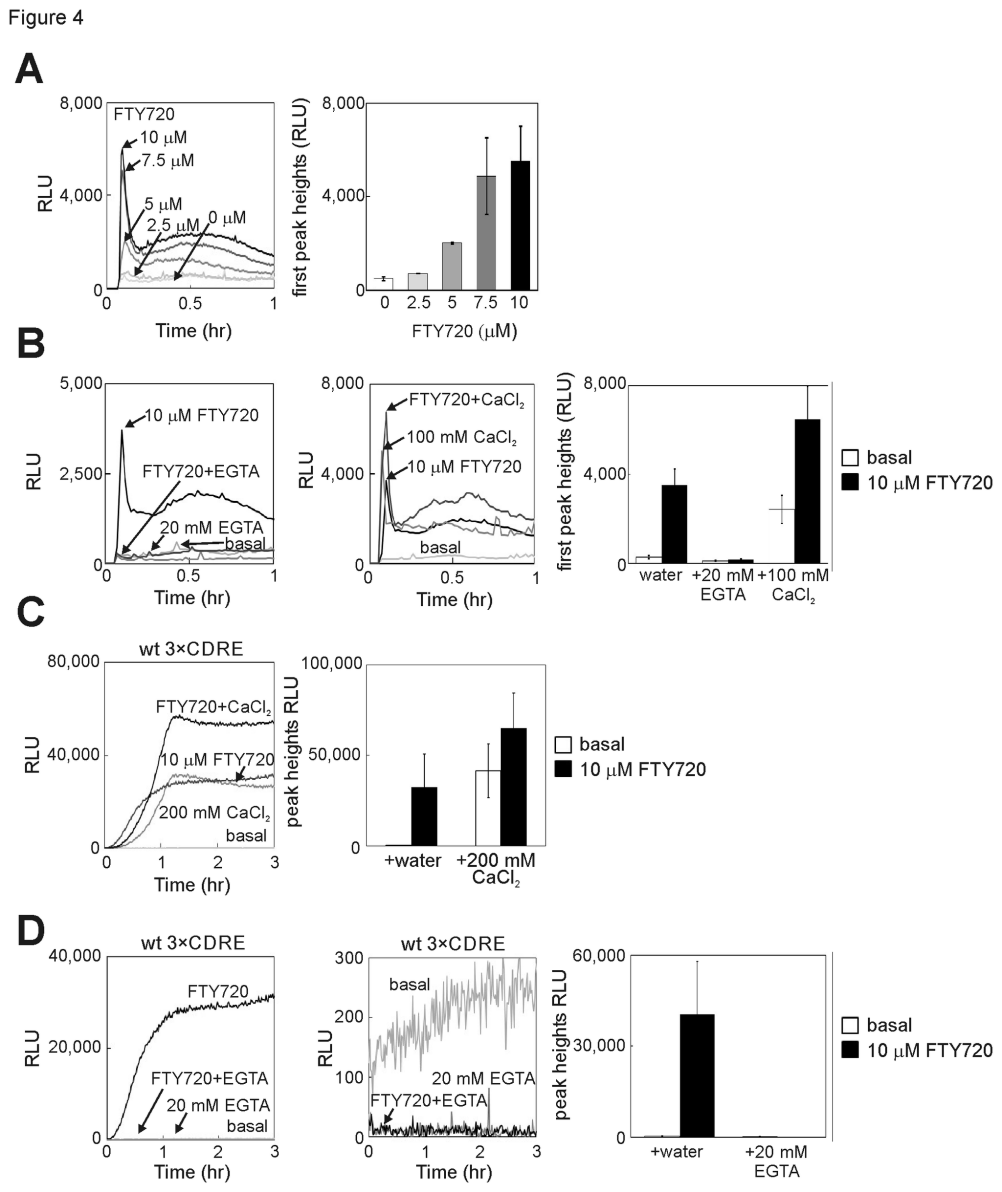
Intracellular Ca^2+^ levels on FTY720 treatment. (A) Left: Monitoring of intracellular Ca^2+^ levels in wild-type (wt) cells harboring *adh1*-GFP-19-AEQ (pKB6892) treated with various concentrations of FTY720 at a time point of 5 min and the luminescence was followed for 1 h. An aequorin assay was performed as described in the Materials and Methods. The data shown are the representative of multiple experiments. Right: Graph shows the average of peak heights from three independent experiments shown in the left column of Figure 4 (A). Bars, SD. (B) Effects of EGTA and CaCl_2_ on the FTY720-induced increase in the cytoplasmic Ca^2+^ level. The experiments were performed as described in Figure 4 (A), except that prior to the addition of FTY720, 20 mM EGTA (left) or 100 mM CaCl_2_ (middle) were added to the EMM medium. Right: The histogram was calculated as described in Figure 4A. (C) (D) Effects of EGTA and CaCl_2_ on the FTY720-induced increase in the calcineurin activity. The experiments were performed as described in Figure 3 (B) with wt 3×CDRE, except that prior to the addition of 10 μM FTY720, 10 mM or 20 mM EGTA or 200 mM CaCl_2_ were added to the EMM medium. The histogram was calculated as described in Figure 3 (B).

Various agents known to cause Ca^2+^ influx reportedly induce calcineurin activation [27]. To provide additional information on the effect of FTY720 on calcineurin signaling, intracellular Ca^2+^ levels upon FTY720 treatment were monitored in fission yeast, using *adh1*-GFP-19-AEQ, which have been established as a sensitive method to monitor the cytoplasmic Ca^2+^ levels [28]. FTY720 induced a dose-dependent increase in cytoplasmic Ca^2+^ levels, which rapidly increased and reached a peak level immediately after the addition of FTY720, rapidly decreased thereafter and exhibited a less pronounced second peak, and then reached a steady state level (Figure 4A). To investigate whether the increase in cytoplasmic Ca^2+^ levels following the addition of FTY720 was due to the influx from the extracellular medium or due to the release from an internal store, the effect of EGTA (extracellular Ca^2+^ **-**chelator) was examined in the same assay. The results demonstrated that the peak responses and increase in cytoplasmic Ca^2+^ levels elicited by FTY720 were almost inhibited by the addition of EGTA to the culture medium (Figure 4B, FTY720 + EGTA). Thus, it is suggested that the increase in cytoplasmic Ca^2+^ levels upon FTY720 treatment is dependent on the influx across the Ca^2+^ machinery involved in Ca^2+^ entry that exists on the plasma membrane. The effect of CaCl_2_ on FTY720-induced Ca^2+^ influx was also examined. As shown in Figure 4B, the addition of 100 mM CaCl_2_ to the medium containing FTY720 markedly increased the peak response compared with that obtained by the addition of 100 mM CaCl_2_ alone, indicating the presence of synergy between CaCl_2_ and FTY720 in stimulating Ca^2+^ influx in fission yeast. 

Similarly, effects of EGTA and CaCl_2_ on FTY720-induced calcineurin activation were examined using the reporter plasmid 3×CDRE::luc(R2.2). The addition of CaCl_2_ induced the additive response of calcineurin activity upon FTY720 treatment (Figure 4C). In addition, the addition of EGTA to the culture medium reduced calcineurin activation by FTY720 (Figure 4D). Therefore, the FTY720-stimulated response activated calcineurin-signaling pathway by a mechanism requiring influx of extracellular Ca^2+^. Thus, these results suggest that FTY720 induced calcineurin activation by stimulating Ca^2+^ influx.

### FTY720 increased the cytoplasmic Ca^2+^ level partly via the Yam8-Cch1 channel complex.

**Figure 5:**
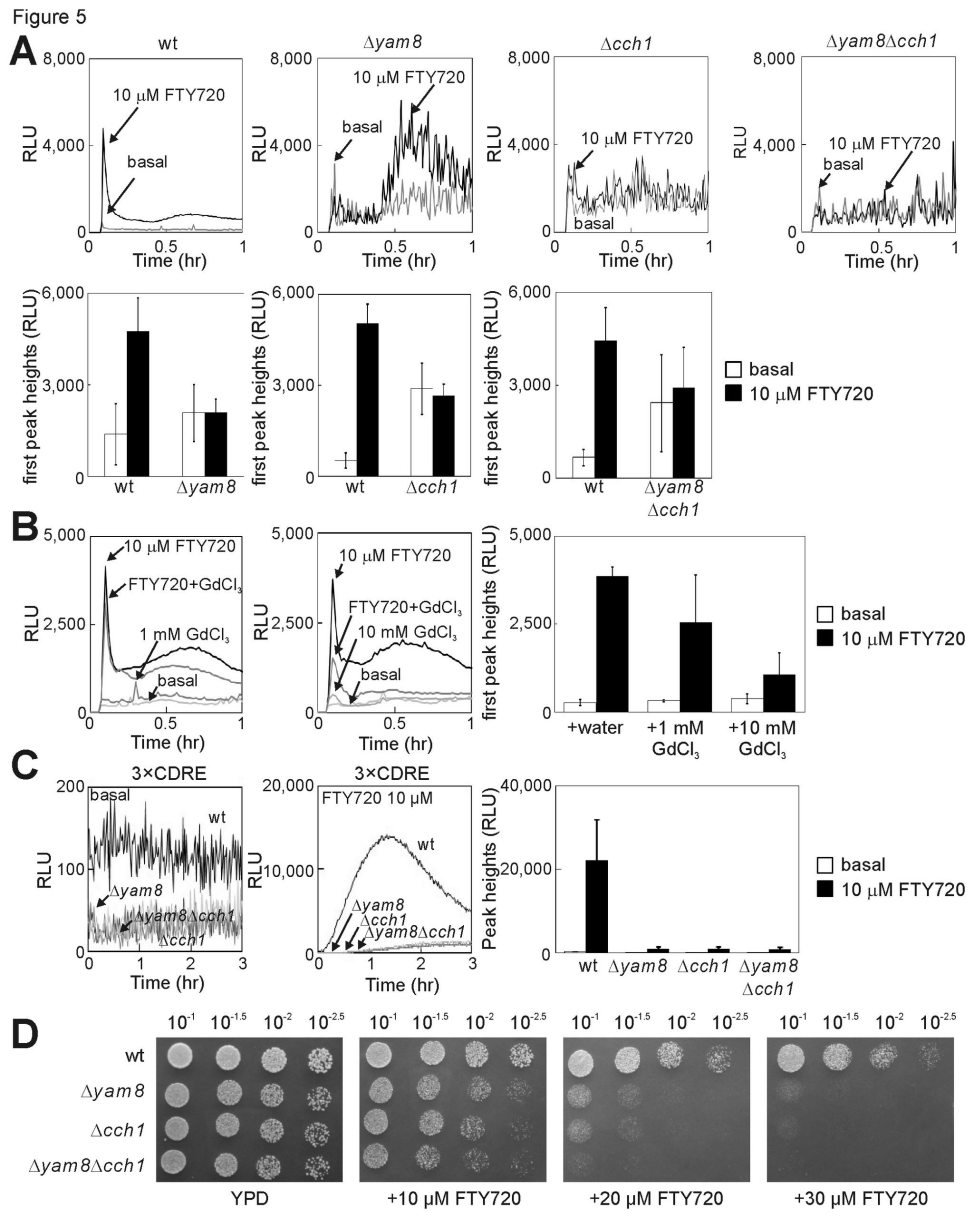
FTY720 stimulates Ca^2+^/calcineurin signaling via the Yam8/Cch1 channel. (A) The wild-type (wt), Δ*yam8*, Δ*cch1*, or Δ*yam8*Δ*cch1* cells harboring pKB6892 (*adh1*-GFP-19-AEQ) were treated with 10 μM FTY720 or vehicle (basal), and the experiments were performed as described in Figure 4 (A). The histogram was calculated as described in Figure 4 (A). Bars, SD. (B) Effects of GdCl_3_ on the FTY720-induced increase in the cytoplasmic Ca^2+^ level. The experiments were performed as described in Figure 4 (A), except that 1 mM or 10 mM of GdCl_3_ were also added to the EMM medium. The histogram was calculated as described in Figure 4A. (C) Deletion of Yam8 or Cch1 reduced markedly calcineurin activation induced by FTY720. The wild-type (wt), Δ*yam8*, Δ*cch1*, or Δ*yam8*Δ*cch1* cells harboring the reporter plasmid (wt 3×CDRE::luc(R2.2)) were monitored as described in the legend of Figure 3(B).　(D) Deletion of Yam8 or Cch1 enhanced the sensitivity to FTY720. A serial dilution assay of the wild-type (wt), Δ*yam8*, Δ*cch1*, and Δ*yam8*Δ*cch1* mutant cells grown in rich YPD medium containing the indicated concentrations of FTY720.

The increase in intracellular Ca^2+^ levels induced by FTY720 was next examined in cells lacking *yam8* ^+^- or *cch1* ^+^-encoding putative subunits of a Ca^2+^ channel, which is reported to regulate Ca^2+^ influx in *S. pombe* [27]. In single and double knockout cells of Yam8 and/or Cch1, the basal cytoplasmic Ca^2+^ level was higher than that in wt cells, consistent with the previous study (Figure 5A) [27,28]. Notably, FTY720 failed to increase the cytoplasmic Ca^2+^ level in these Ca^2+^ channel mutant cells (Figure 5A). 

In addition, the effect of a Ca^2+^ channel blocker, Gadolinium (III) chloride (GdCl_3_), on Ca^2+^ influx induced by FTY720 was examined. GdCl_3_ reduced the peak response of Ca^2+^ levels upon FTY720 treatment in a dose-dependent manner (Figure 5B), consistent with a previous report that Ca^2+^ currents mediated by Mid1, a homologue of Yam8 in budding yeast, expressed in Chinese hamster ovary cells were inhibited by Gd^3+^ [33]. Because Gd^3+^ is not efficiently transported across the plasma membrane, these data suggest that the increase in cytoplasmic Ca^2+^ levels induced by FTY720 depends on Ca^2+^ influx from the extracellular medium.

Furthermore, FTY720 failed to activate calcineurin in these Ca^2+^ channel mutant cells because the response of the 3×CDRE reporter stimulated by the addition of FTY720 was reduced markedly in these mutant cells (Figure 5C). These results suggest that the effect of FTY720 on calcineurin signaling is mediated at least in part by the activation of the Yam8-Cch1 channel complex and the resultant Ca^2+^ influx. Interestingly, single and double knockout cells of Yam8 and/or Cch1 exhibited enhanced sensitivity to FTY720 because these mutant cells barely grew in the presence of 30 μM FTY720, whereas the wt cells grew well (Figure 5D). The hypersensitivity to FTY720 may reflect defective Ca^2+^ homeostasis in these mutant cells.

**Figure 6:**
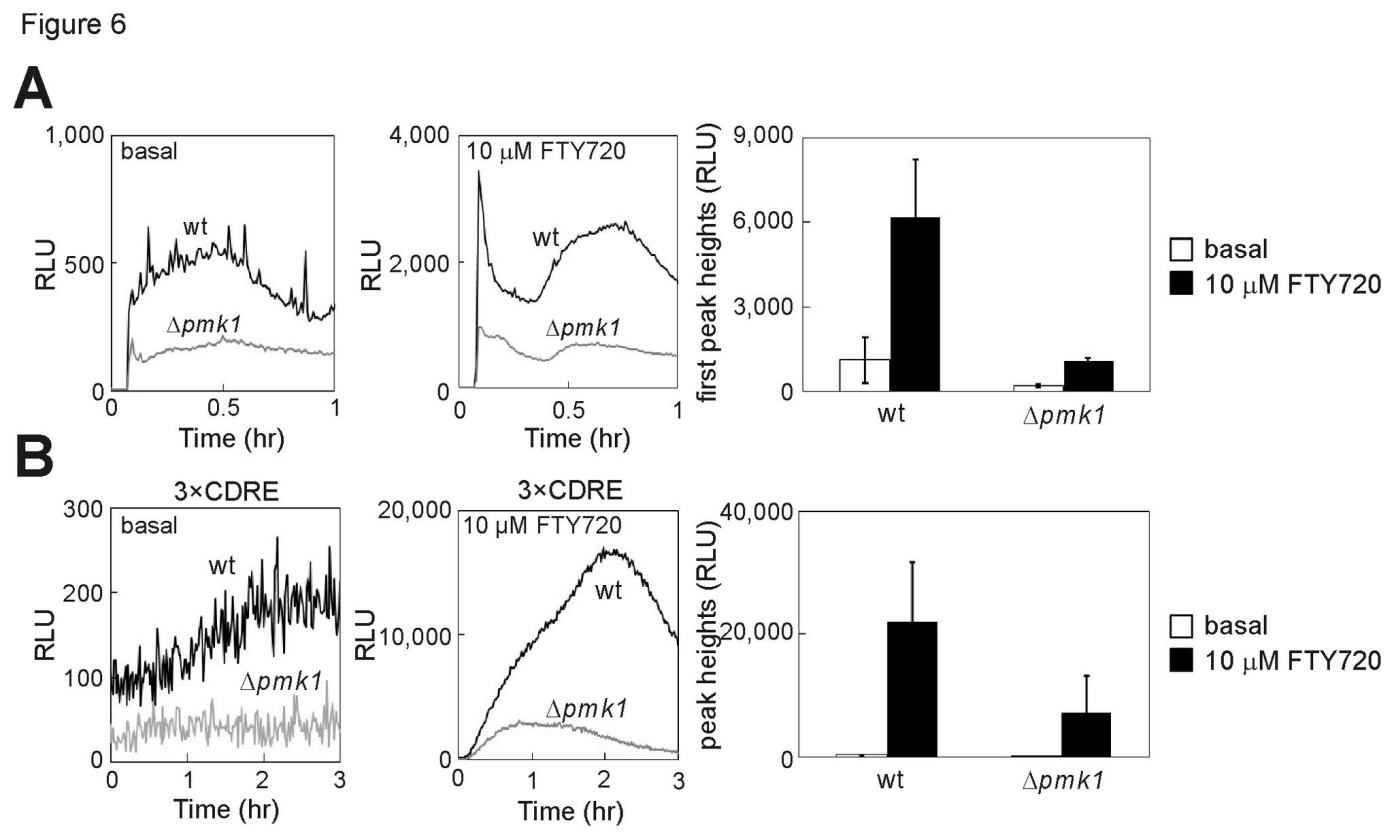
Pmk1 MAP kinase activated the FTY720-induced Ca^2+^ influx. (A) Knockout of Pmk1 MAP kinase suppressed the FTY720-induced Ca^2+^ influx. WT and Δ*pmk1* cells were monitored as described in the legend of Figure 4 (A). (B) Knockout of Pmk1 MAP kinase suppressed the FTY720-induced calcineurin signaling. WT and Δ*pmk1* cells were monitored as described in the legend of Figure 3 (B).

The effect of the Pmk1 MAPK pathway was also examined because this pathway has been reported to positively regulate the activation of the Yam8/Cch1 channel complex in both yeasts [27]. As shown in Figure 6A, in Pmk1-null cells, the basal cytoplasmic Ca^2+^ level was very low compared with wt cells, and the Ca^2+^ increase induced by the addition of FTY720 was reduced markedly (Figure 6A). In addition, the FTY720-mediated calcineurin activation was reduced markedly by the deletion of *pmk1* ^+^ (Figure 6B).

### The effect of FTY720-P on Ca^2+^/calcineurin signaling.

**Figure 7:**
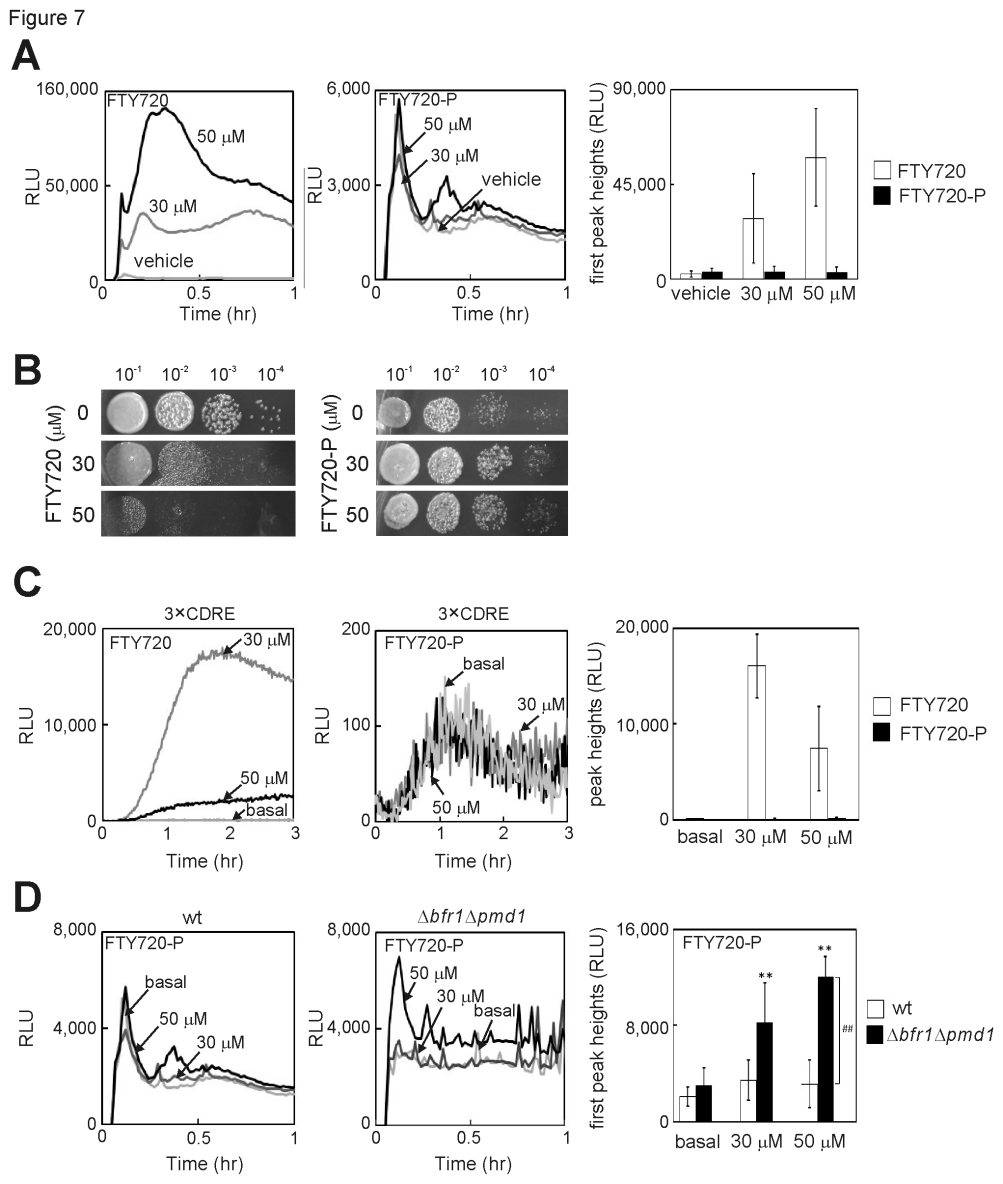
Phosphorylated FTY720 (FTY720-P) failed to stimulate Ca^2+^/calcineurin signaling. (A) Effect of FTY720 and FTY720-P on intracellular Ca^2+^ levels. The wild-type cells harboring *adh1*-GFP-19-AEQ were treated with indicated concentrations of FTY720 or FTY720-P, and experiments were performed as described in Figure 4(A). Ethanol containing NaOH was used as vehicle (Materials and Methods). The histogram was calculated as described in Figure 4 (A). Bars, SD. (B) A serial dilution assay of the wild-type (wt) cells grown in rich YPD medium containing the indicated concentrations of FTY720 or FTY720-P. (C) Left: Real-time monitoring of calcineurin activity in living cells stimulated by FTY720 or FTY720-P. Wild-type cells harboring the multicopy plasmid (wt 3xCDRE::luc(R2.2)) reporter vector, pKB5723 (wt 3xCDRE) were treated as described in Figure 3(B). The data shown are representative of multiple experiments. Right: Graph shows the peak heights of 3xCDRE::luc(R2.2) reporter activity. The data were averaged from three independent experiments. Bars, SD. (D) The effect of FTY720-P induced rise of intracellular Ca^2+^ levels in ABC transporter knockout cells. The wt or Δ*bfr1*Δ*pmd1* cells harboring *adh1*-GFP-19-AEQ were treated with indicated concentrations of FTY720-P, and experiments were performed as described in Figure 4(A). ** p<0.01; Significantly different from vehicle (using two-way ANOVA). ## p<0.01; Significantly different from wild-type cells (using Williams’ test).

We wanted to determine whether the phosphorylated form of FTY720 (FTY720-P) also stimulates Ca^2+^ signaling. Notably, unlike FTY720, FTY720-P failed to induce Ca^2+^ influx because the addition of up to 50 μM FTY720-P did not affect the intracellular Ca^2+^ concentration (Figure 7A, FTY720-P). In contrast, nonphosphorylated FTY720 effectively stimulated Ca^2+^ influx in a dose-dependent manner (Figure 7A, FTY720). It should be noted that the pattern of the first sharp peak of Figure 7A (left panel) and Figure 4A was reproducibly different and the possible reasons for the different pattern may be due to the difference in the vehicles used in each experiment (Materials and Methods). This was further confirmed by the experiments comparing the pattern obtained with the vehicle used in Figure 4A (water) and the vehicle used in Figure 7A (ethanol containing NaOH for the insolubility of FTY720-P in water) (Figure S1). In addition, the inhibitory effect on cell growth was not observed in the medium containing 50 μM of FTY720-P, whereas no colonies were formed in the presence of the same concentration of nonphosphorylated FTY720 (Figure 7B). Finally, the addition of up to 50 μM FTY720-P did not affect the CDRE responses (Figure 7C). In addition, FTY720-P did not affect the intracellular localization of Prz1 as compared with vehicle alone (Figure S2). 

We sought to take into account the possibility that the cellular penetrance of FTY720-P might be limited by the S. *pombe* cell wall as well as the possibility that the compound might be more rapidly metabolized in *S. pombe* cells than in animal cells. Because FTY720 was reported to be transported across cellular membranes with ATP-binding cassette (ABC) transporter family members [34-36], the observed lack of effect of FTY720-P on Ca^2+^ signaling could be due to rapid export of the compound from the cells. We then tested the intracellular Ca^2+^ levels induced by FTY720-P in cells lacking both *bfr1* ^*+*^ and *pmd1* ^*+*^, encoding two major *S. pombe* drug-efflux transporters, which were involved in multidrug resistance [37,38]. As shown above, the addition of FTY720-P did not affect the intracellular Ca^2+^ levels in wild-type cells (Figure 7A). In contrast, in Δ*bfr1*Δ*pmd1* null cells, the intracellular Ca^2+^ levels were significantly higher than wt cells in the absence and the presence of FTY720-P (Figure 7D). Data were also statistically analyzed using Williams’ test and the analysis indicated that the increase in intracellular Ca^2+^ concentrations upon FTY720 treatment in Δ*bfr1*Δ*pmd1* null cells were statistically significant, with a P-value of < 0.01 (**) and that the difference in intracellular Ca^2+^ levels upon FTY720 stimuli (50 μM) between wt and Δ*bfr1*Δ*pmd1* null cells were significant, with a P-value of < 0.01 (##) (Figure 7D; right panel). This result suggested the possibility that FTY720-P may poorly enter the cells and due to subsequent export of the drug by Bfr1 and Pmd1 resulted in the apparent loss of biological activity with respect to its effect on the intracellular Ca^2+^ concentration and calcineurin-mediated transcriptional activity.

## Discussion

Here, we used the fission yeast model system to analyze the effect of FTY720 on Ca^2+^/calcineurin signaling and presented several lines of evidence that FTY720 can stimulate Ca^2+^ influx largely via Cch1/Yam8, resulting in the elevation of cytoplasmic Ca^2+^ levels and activation of calcineurin signaling pathway. 

Because the fission yeast genome does not express structural homologues of S1P receptors in mammals, the observed responses induced by FTY720 could be mediated independently of S1P receptors. A number of recent studies suggest that FTY720 exerts various biological functions, including anti-cancer activities, and these effects other than immune modulation can be mediated independently of S1P receptors. Furthermore, while the functions of FTY720-P as a immunomodulator are carried out at nanomolar concentrations, a higher concentration of non-phosphorylated FTY720 were required to exert its biological activities such as inducing apoptosis[4,9,10,29-32,39]. The candidate cellular targets of FTY720 include cPLA_2_ [12], 14-3-3 [40], PKC [41], and PP2A [42]. Notably, FTY720 also increases the intracellular concentration of calcium ions and induces apoptosis in HL-60 [10], and although the involvement of PLC was suggested, cellular targets and the intracellular action mechanism of FTY720 regarding these effects remain to be fully elucidated. Here, we showed that the biological effects of FTY720 can be partly explained by Yam8/Cch1 channel and Ca^2+^/calcineurin signaling activation. Our previous study suggested that the persistent increase in the cytoplasmic Ca^2+^ level causes cell death due to apoptotic process in *S. pombe* [28]. Therefore, it would be intriguing if pro-apoptotic properties of FTY720 in mammals may also involve hyperactivation of Ca^2+^/calcineurin signaling pathway in higher eucaryotes, which is consistent with the notion that Ca^2+^/calcineurin signaling induces apoptosis.

Fission yeast cells also have the homolog of sphingoid long-chain base (LCB) kinase, pSPHK1 (accession no. T38776) that catalyzes the phosphorylation of LCBs to form LCB 1-phosphate in mammals [43]. However, pSPHK1 knockout cells were not severely sensitive to FTY720 in *S. pombe* (data not shown), as previously reported in *Saccharomyces cerevisiae* [19,20], which suggests that FTY720-P may play a role in FTY720 mediated effects. Therefore, our observation that FTY720-P does not stimulate calcium flux and calcineurin activation in fission yeast may only be true of exogenously applied FTY720-P. Intracellularly produced FTY720-P would not be subject to the same transport barriers as exogenous FTY720-P and may have biological activity. 

The involvement of the Ca^2+^ channel protein Yam8/Cch1 underscores the existence of a specific mechanism for FTY720-dependent calcium signaling in fission yeast. Whether FTY720 can traverse the yeast cell wall and plasma membrane to act intracellularly, or affects cell integrity, thereby activating Yam8/Cch1 channel remains unknown. However, we favor the former possibility based on the lipophilic nature of FTY720, as well as our findings that an anti-fungal agent such as miconazole, or detergent such as 1% Tween 20, or an ER-stress-inducing compound DTT, failed to induce Ca^2+^ influx as efficiently as FTY720 did (our unpublished results). In addition, in the presence of extracellular Ca^2+^-chelators such as EGTA, FTY720 was unable to stimulate Ca^2+^ influx. Therefore, no evidence for FTY720-stimulated Ca^2+^ release from the internal store has been obtained in fission yeast thus far. It should be noted however, that EGTA addition failed to rescue the growth inhibition of wild-type cells by FTY720 (Figure S3). In addition, the addition of CaCl_2_ (up to 100 mM) failed to rescue the growth inhibition of Δ*yam8*, Δ*cch1*, Δ*yam8*Δ*cch1* cells, thus suggesting that FTY720-induced cell growth defect may not be explained only by activation of Ca^2+^/calcineurin signaling, and may instead represent a broader effect. We therefore hypothesized that although the disturbance of appropriate regulation of the Ca^2+^/calcineurin signaling resulted in increased sensitivity of fission yeast proliferation to adapt to physiological responses induced by FTY720, and that Yam8/Cch1 plays an important role on calcineurin activation induced by FTY720, it may not support a direct connection between calcineurin activity and inhibition of proliferation. Because fission yeast cells also have the homolog of sphingoid long-chain base (LCB) kinase, pSPHK1 (accession no. T38776) that catalyzes the phosphorylation of LCBs to form LCB 1-phosphate in mammals [43], it would be intriguing if these kinases may be involved in the FTY720-mdiated Ca^2+^/calcineurin signaling in fission yeast.

The Ca^2+^/calcineurin signaling pathway plays an important role in various cellular functions, including immune regulation, cardiac hypertrophy and apoptosis, and FK506 and CsA are specific inhibitors of calcineurin both in humans and yeasts. Notably, the findings presented in this study suggested the cross-talk between FTY720-mediated and FK506-regulated signaling pathway, both of which are involved in immune regulation. In clinical organ transplantations, FK506-based combination therapy with other immunosuppressants is widely used to reduce the side effects of individual drugs. Notably, the combination therapy of FTY720 with CsA or FK506 has a marked prolonging effect on allograft survival as compared with the monotherapy of FTY720, CsA, or FK506 [2]. Our findings of FTY720-stimulated calcineurin activation may underlie the mechanism of synergistic effect of FTY720 in combination with calcineurin inhibitors. Because FTY720-stimulated calcineurin hyperactivation could result in the activation of the immune system, and may thus counteract the immune modulation of FTY720, FK506 treatment can inhibit calcineurin hyperactivation, thus inducing synergistic effect. A future chemical biology screen to search for mutants with altered sensitivity to FTY720 using fission yeast model system may be applicable to identify new factors required for FTY720-mediated Ca^2+^ signaling pathway. 

## Supporting Information

Figure S1 **Effect of vehicle on the basal intracellular Ca^2+^** **levels**. Left panel: The wild-type cells harboring *adh1*-GFP-19-AEQ were treated with water and ethanol containing NaOH, and experiments were performed as described in Figure 4(A). Right panel: The histogram was calculated as described in Figure 4 (A). Bars, SD.
(TIF)

Figure S2 **Effect of FTY720-P on the intracellular localization of GFP-Prz1.** Translocation of GFP-Prz1 to the nucleus is induced by FTY720 addition, but not by FTY720-P. Wild-type cells expressing GFP-Prz1 were grown in EMM medium at 27°C and analyzed by fluorescence microscopy as described in Figure 3(A). The bar indicates 10 μm.
(TIF)

Figure S3 **The effect of Ca^2+^ or EGTA on inhibition of proliferation by FTY720 in various strains.** A serial dilution assay of the wild-type (wt), Δ*yam8*, Δ*cch1*, and Δ*yam8*Δ*cch1* cells grown in rich YPD medium containing the indicated concentrations of FTY720, CaCl_2_ and EGTA.
(TIF)
