# Supplementary figures and images for: Fingolimod (FTY720) Stimulates Ca2+/Calcineurin Signaling in Fission Yeast
Source: PLoS One. 2013 Dec 3;8(12):e81907. doi: 10.1371/journal.pone.0081907 (PMC3849299; doi:10.1371/journal.pone.0081907)

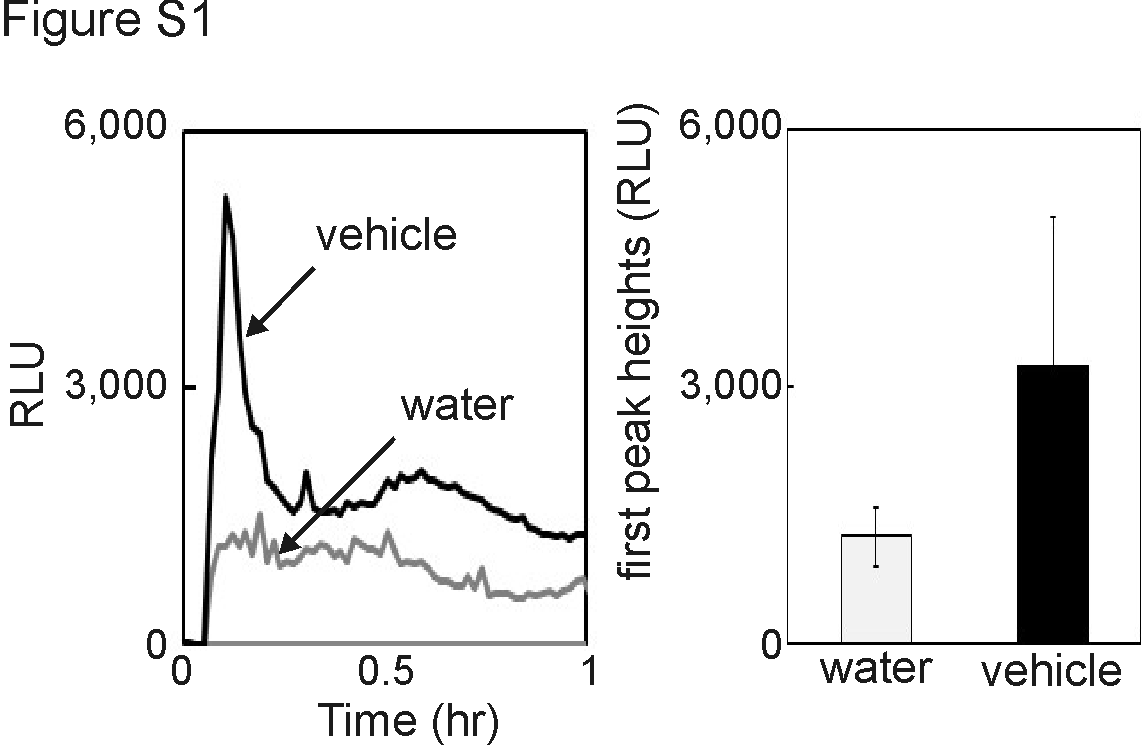

Supplement: Figure S1 — Effect of vehicle on the basal intracellular Ca2+ levels. Left panel: The wild-type cells harboring adh1-GFP-19-AEQ were treated with water and ethanol containing NaOH, and experiments were performed as described in Figure 4(A). Right panel: The histogram was calculated as described in Figure 4 (A). Bars, SD. (TIF) [file pone.0081907.s001.tif]

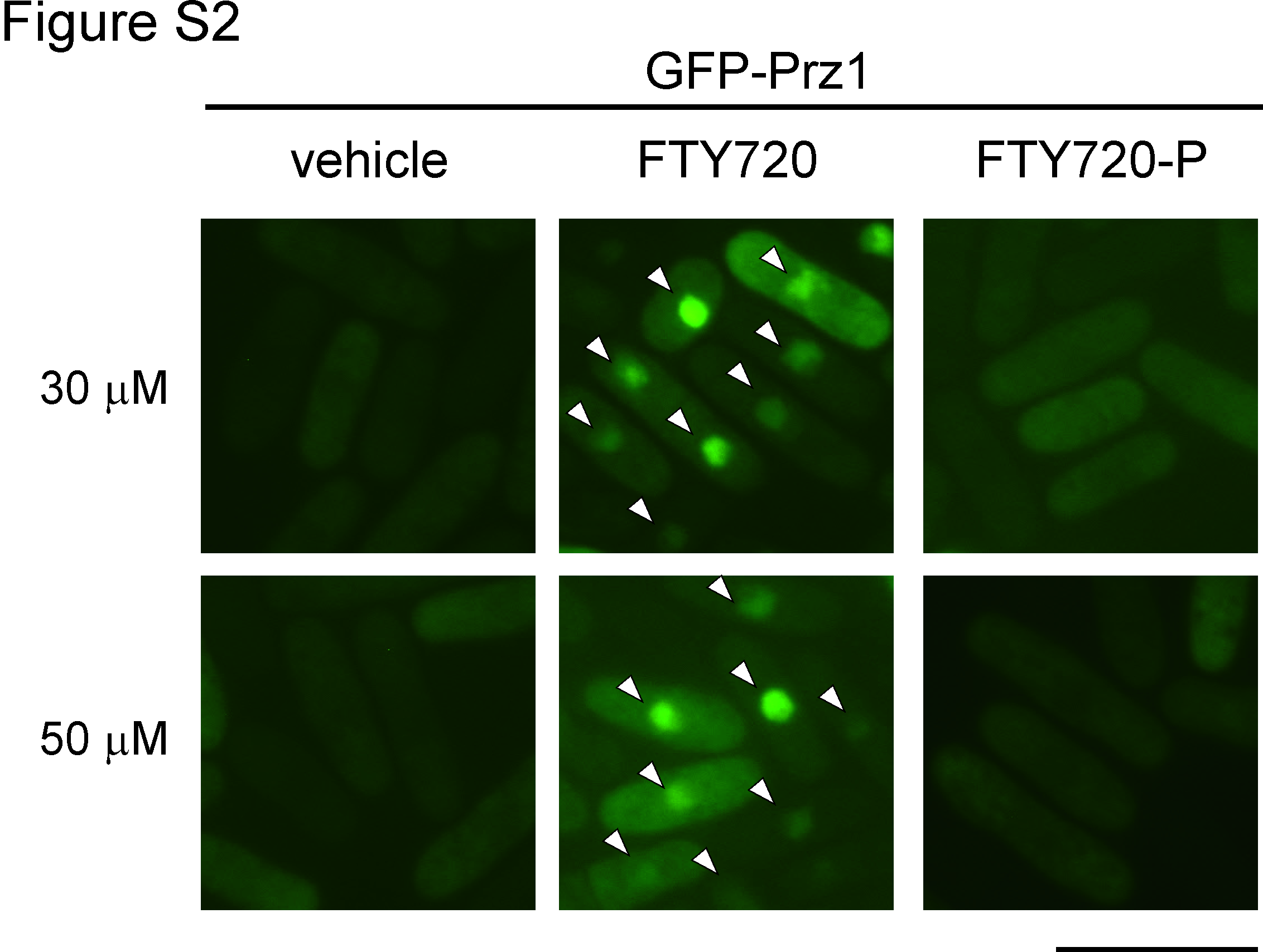

Supplement: Figure S2 — Effect of FTY720-P on the intracellular localization of GFP-Prz1. Translocation of GFP-Prz1 to the nucleus is induced by FTY720 addition, but not by FTY720-P. Wild-type cells expressing GFP-Prz1 were grown in EMM medium at 27°C and analyzed by fluorescence microscopy as described in Figure 3(A). The bar indicates 10 μm. (TIF) [file pone.0081907.s002.tif]

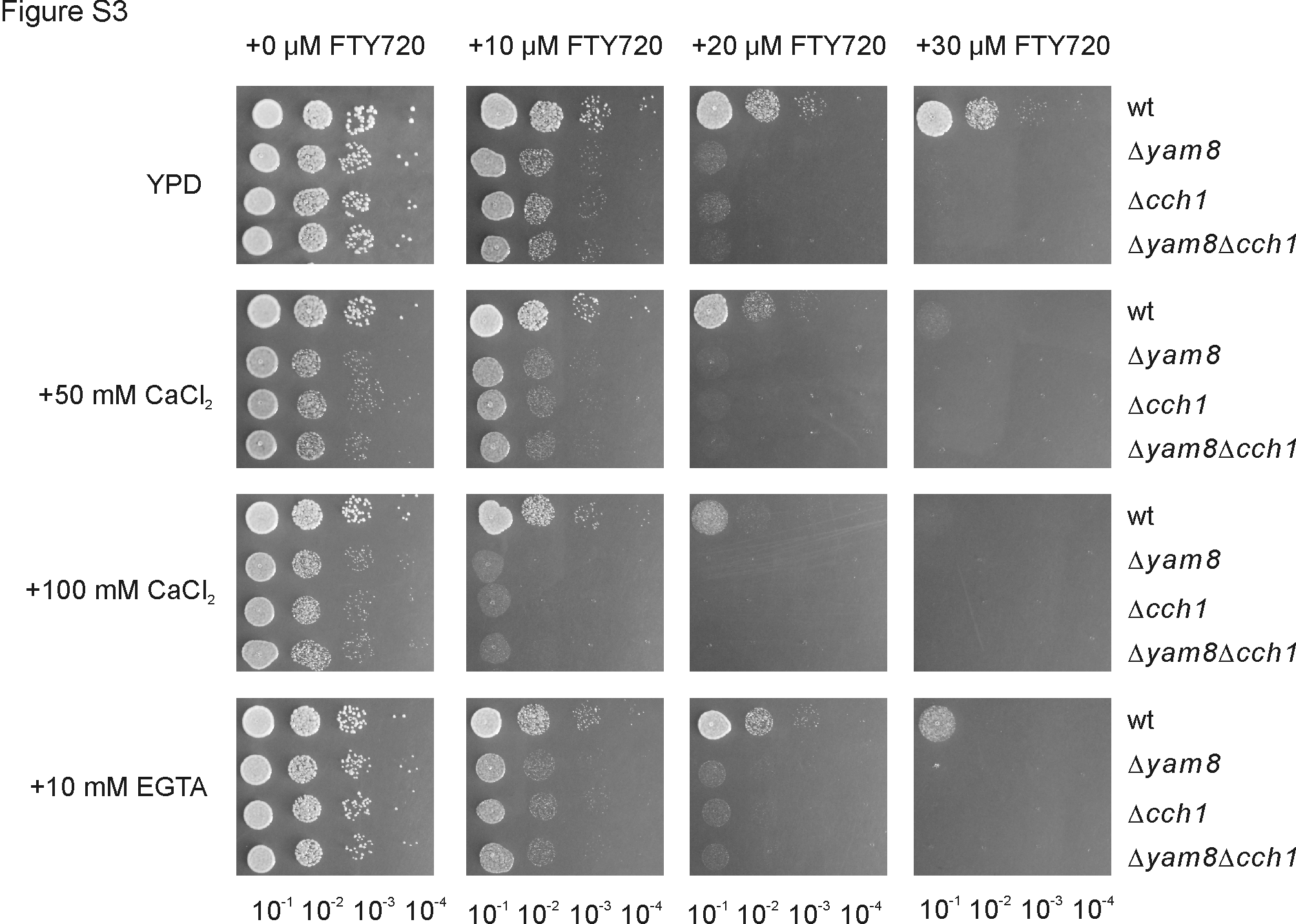

Supplement: Figure S3 — The effect of Ca2+ or EGTA on inhibition of proliferation by FTY720 in various strains. A serial dilution assay of the wild-type (wt), Δyam8, Δcch1, and Δyam8Δcch1 cells grown in rich YPD medium containing the indicated concentrations of FTY720, CaCl2 and EGTA. (TIF) [file pone.0081907.s003.tif]
